# Immunogenicity of virus-like particle vaccine candidates against SARS-CoV-2 infection

**DOI:** 10.1099/acmi.0.000925.v3

**Published:** 2025-02-17

**Authors:** Hai Trong Nguyen, Ravendra Garg, Andrea Kroeker, Volker Gerdts, Darryl Falzarano, Qiang Liu

**Affiliations:** 1 Vaccine and Infectious Disease Organization (VIDO), University of Saskatchewan, Saskatoon, Saskatchewan, Canada; 2 Department of Veterinary Microbiology, Western College of Veterinary Medicine, University of Saskatchewan, Saskatoon, Saskatchewan, Canada; 3 School of Public Health, University of Saskatchewan, Saskatoon, Saskatchewan, Canada

**Keywords:** neutralizing antibody, recombinant baculovirus, severe acute respiratory syndrome coronavirus 2 (SARS-CoV-2), vaccine, variants of concern, virus-like particles

## Abstract

Severe acute respiratory syndrome coronavirus 2 (SARS-CoV-2) continues to evolve, potentially leading to variants of concern that could become more transmissible, resist treatment, evade host immunity and reduce the effectiveness of currently available vaccines. Improved vaccines are still required as vaccination remains the most effective strategy against this virus. We have produced two SARS-CoV-2 virus-like particles (VLPs) using a baculovirus BacMam expression platform and examined their immunogenicity in mice. VLP1 contains the spike protein from the Wuhan strain, whereas VLP2 contains that of an Omicron variant. Mice immunized with VLP1 and boosted with VLP2 developed significantly higher antibodies in the sera, as well as higher numbers of IFN-γ secreting cells than the control group. Furthermore, both VLPs induced virus-neutralizing antibodies against Wuhan and Omicron variants. In conclusion, VLPs have the potential for the development of a safe and effective vaccine against SARS-CoV-2 variants.

## Data Summary

Plasmid sequences were submitted to GenBank (accession numbers: PV053552 and PV053553).

## Introduction

Severe acute respiratory syndrome coronavirus 2 (SARS-CoV-2) is a novel coronavirus responsible for the coronavirus disease 2019 (COVID-19) pandemic which began in late 2019 [1,2]. SARS-CoV-2 has a high transmission rate, largely spread through respiratory droplets, and can cause various symptoms, from mild to severe, such as fever, cough and difficulty breathing [3]. Several authorized COVID-19 vaccines have been available and help prevent COVID-19 infection and reduce the severity of illness. Variants of the virus have emerged, with enhanced virulence, transmissibility and reduced response to current diagnostics, vaccines and therapeutics, posing challenges to global efforts. COVID-19 vaccines have shown varying degrees of effectiveness against different virus variants [4,5]. More effective, safer and broad-spectrum vaccines are still required to mitigate SARS-CoV-2 infection since immunization remains critical in controlling its spread.

Virus-like particles (VLPs) are molecular self-assembled structures that resemble viruses but are non-infectious because they lack genetic material [6,8]. VLPs are made up of viral structural proteins that self-assemble to mimic real viruses and display their surface antigens [9]. VLP-based vaccines, such as those against hepatitis B and human papillomavirus, are highly effective and safe, as they can stimulate a robust humoral and cellular immune response without the risk of causing disease [9,10]. In this study, we have developed recombinant baculovirus-based SARS-CoV-2 VLPs expressing spike proteins of the ancestral Wuhan or an Omicron variant and evaluated the immunogenicity of the SARS-CoV-2 VLP antigen in mice upon prime and boost immunization.

## Methods

### Cells and culture media

Vero76, HEK293, HEK293T and ACE2-expressing HEK293 cells were cultured and maintained in Dulbecco’s Modified Eagle Medium (DMEM) (11965–092, Gibco) supplemented with 10% (vol/vol) foetal bovine serum (12107C, Sigma-Aldrich) and 100 units penicillin and 100 µg streptomycin ml^−1^ (P4333-100ML, MilliporeSigma) (designated as D10 medium) at 37℃ in 5% CO_2_ atmosphere.

### Plasmid construction

The coding sequences for spike, membrane, envelope and nucleocapsid proteins of SARS-CoV-2 original strain Wuhan (GenBank accession number: NC_045512) were codon optimized for mammalian expression and synthesized by Bio Basic Inc., Canada. The S gene was first inserted into pFastBacTriple1 as this vector has great capacity for carrying large and multiple DNA fragments and could be used as a versatile system to produce coronavirus VLP in mammalian cells. Subsequently, M and E expression cassettes amplified by PCR with [1] Rsr2-CMV-F and CMV-Cov2-M-R [2]; CMV-Cov2-M-F and pP10-CMV-R [3], pP10-CMV-F and CMV-Cov2-E-R [4] and CMV-Cov2-E-F and MfeI-Cov2-E-R primers and N expression cassette amplified by PCR with [1] XhoI-CMV-F and CMV-Cov2-N-R [2], CMV-Cov2-N-F and Cov2-N-SV40-R and [3] Cov2-N-SV40-F and NheI-SV40-R primers were then assembled into the S-expressing plasmid using GenBuilderᵀᴹ Plus Cloning Kit (L00744-50, GenScript) to generate a SARS-CoV-2 VLP-expressing plasmid assigned as pSARS-CoV-2-VLP1 (GenBank accession number: PV053552). Each SARS-CoV-2 structural protein gene was placed downstream of a cytomegalovirus (CMV) promoter and followed by a poly A signal sequence. HSV TK poly A signal sequence was inserted downstream of the S and M genes, while SV40 poly A signal sequence was cloned downstream of the E and N genes. To generate a SARS-CoV-2 VLP containing spike protein of the Omicron B.1.1.529 variant (EPI_ISL_6699768), the Wuhan S protein gene was replaced by the synthesized Omicron S coding sequence (assigned as pSARS-CoV-2-VLP2; GenBank accession number: PV053553). Phusion™ High-Fidelity DNA Polymerase (F530L, Thermo Fisher Scientific) was used for PCR amplification of desired DNA sequences. Primers for PCR for plasmid construction are listed in Table 1. All constructed plasmids were verified by PCR targeting the insertion junctions, restriction enzyme digestion and DNA sequencing.

**Table 1.** Primers for construction of SARS-CoV-2 VLP-expressing plasmids

No.	Primers	Sequences (5′-3′)
1	Rsr2-CMV-F	cataccgtcccaccatcgggcgcggatcccggtccgACATTGATTATTGACTAGTTATT
2	CMV-Cov2-M-R	gctatcggccatggtggcGCCAGTAAGCAGTGGGTTCTC
3	CMV-Cov2-M-F	ctgcttactggcgccaccATGGCCGATAGCAACGGGAC
4	pP10-CMV-R	gtcaataatcaatgtGGTGATATCGTGTCGGGCC
5	pP10-CMV-F	cgacacgatatcaccACATTGATTATTGACTAGTTATT
6	CMV-Cov2-E-R	gctgtacatggtggcGCCAGTAAGCAGTGGGTTCTC
7	CMV-Cov2-E-F	gcttactggcgccaccATGTACAGCTTCGTGTCCGAGG
8	MfeI-Cov2-E-R	ccattataagctgcaataaacaagttaacaacaaCAATTGCATTCATTTTATGTTTCAGG
9	XhoI-CMV-F	ctgtaaattacattttatttacaatcactcgacgaagacttgatcacccgggatctcgagACATTGATTATTGACTAGTT
10	CMV-Cov2-N-R	ctggggcccgttatcactcatggtggcGCCAGTAAGCAGTGGGTTCTC
11	CMV-Cov2-N-F	gagaacccactgcttactggcgccaccATGAGTGATAACGGGCCCCAG
12	Cov2-N-SV40-R	gacaagcttggtaccgcatgctcaGGCCTGTGTGCTATCAGCGC
13	Cov2-N-SV40-F	gcgctgatagcacacaggcctgaGCATGCGGTACCAAGCTTGTC
14	NheI-SV40-R	ccgtaattgattactattaataactagtcaataatcaatgtgctagcCTCAAGCAGTGATCAGATCC

### Generation and purification of SARS-CoV-2 VLPs

To produce SARS-CoV-2 VLPs, HEK293 cells seeded overnight in T75 flasks at 60–70% confluence were transfected with 20 µg per flask of the pSARS-CoV-2-VLP1 or pSARS-CoV-2-VLP2 plasmids using JetPEI transfection reagent (101000020, Polyplus). The transfection mixes were replaced with fresh culture medium at 4 h post-transfection (hpt). Cell culture medium containing SARS-CoV-2 VLPs was harvested at 48 and 72 hpt, and then fresh medium was added to the cells. At 96 hpt, the cell culture medium was last harvested, pooled with the ones collected at earlier time points and centrifuged at 1000 ***g*** for 10 min at 4℃ to remove the cell debris. The transfected cells were collected at 96 hpt, suspended in PBS supplemented with a protease inhibitor cocktail, undergone three freeze-thaw cycles and clarified by centrifugation at 1000 ***g*** for 10 min at 4℃.

SARS-CoV-2 VLPs were purified as previously described with minor modifications [11,12]. Specifically, clarified VLP-containing supernatant were filtered through a 0.45-µm filter and layered over a 20% sucrose cushion in Tris/Saline/EDTA (TSE) buffer (0.02 M Tris, 0.15 M sodium chloride and 0.002 M EDTA disodium salt; pH 8.0) and centrifuged at 100 000 ***g*** for 3 h at 4℃ in a SW32 rotor using Beckman Optima-L100 XP Ultracentrifuge. The supernatant was carefully discarded, and the VLP-containing pellet was gently resuspended in PBS (pH 7.3) and stored at −80℃ for further analysis. The total protein concentration of the VLP samples was determined by Bradford protein assay (Bio-Rad).

### Western blotting

SARS-CoV-2 VLPs prepared in SDS sample buffer were subjected to SDS-PAGE and transferred onto nitrocellulose membranes (Millipore). The membranes were then blocked with 3% BSA in PBST (PBS+0.1% Tween 20; pH 7.3) at room temperature (RT) for 1 h and prior to being incubated with a primary antibody at 4℃ overnight. Rabbit anti-SARS-CoV-2 spike protein S1/S2 antibody (PA5-112048, Thermo Fisher Scientific), rabbit anti-SARS-CoV membrane (M) protein antibody (100–401 A55, Rockland), rabbit anti-SARS-CoV-2 envelope (E) protein polyclonal antibody (SARS-COV2-E-101AP, Thermo Fisher Scientific) and rabbit SARS-CoV-2 N protein antisera (produced in house [13]) were used as primary antibodies. Subsequently, IRDye 680 goat anti-rabbit IgG (926–68071, Li-Cor Biosciences) secondary antibody (diluted 1:10 000) was added and incubated for 1 h at RT. The membranes were washed five times with PBST after each incubation step and then scanned with the Odyssey CLx Imaging System (Li-Cor Biosciences).

### Transmission electron microscope imaging

Purified SARS-CoV-2 VLPs were subjected to negative staining and examined by transmission electron microscopy. Specifically, a 400-mesh copper grid (Electron Microscopy Sciences, Hatfield, PA, USA) coated with formvar and carbon was placed on a drop of the purified VLP sample and incubated at RT for 1–2 min for the particles to adhere, prior to being transferred to a droplet of water for 20 s. Subsequently, the grid was placed on a drop of 0.5% PTA stain solution for 1 min, wicked away the excessive stain and dried. The grid was then analyzed by using a transmission electron microscope, HT7700 (Hitachi High-Tech, Tokyo, Japan), and images were acquired through a CCD camera, XR16 (AMT, Woburn, MA) at the WCVM Imaging Centre (University of Saskatchewan, Canada).

### Immunization

To evaluate the immunogenicity of the SARS-CoV-2 VLPs, female BALB/c mice (aged 5–6 weeks, Charles River Laboratories) were vaccinated intramuscularly with 2.5 µg VLP1 and then boosted with 2.5 µg VLP2 3 weeks later. Animal procedures were approved by the Animal Research Ethics Board, University of Saskatchewan (Protocol # 2020–0016). Animals were euthanized 3 weeks after the second vaccination, and their serum and spleen were collected for immunoassays.

### ELISA

Clear round-bottom Immuno 96-well plates (1424579, Thermo Fisher Scientific) were coated with 100 µl of 2 µg ml^−1^ SARS-CoV-2 N protein produced in-house [13], Wuhan S1 and B1.1.529 (Omicron) S1 (40591-V08H41-100, Sino Biological) at 4℃ overnight. The plates were blocked with 150 µl of 1% bovine serum albumin (A7906-100g, Sigma-Aldrich) in TBST (Tris-buffered saline+0.1% Tween 20; pH 7.5) for 1 h. Subsequently, 100 µl of twofold diluted mouse serum at an initial dilution of 1:200 was added to the coated wells and incubated for 2 h. Then, Biotin-labelled goat anti-mouse IgG1 (A10519, Thermo Fisher Scientific) or Biotin-labelled goat anti-mouse IgG2a (M32315, Thermo Fisher Scientific) was added and incubated for 1 h. Subsequently, the wells were incubated with streptavidin alkaline phosphatase (106050084; Jackson ImmunoResearch Laboratories) for 1 h. Incubation was done at RT, and the plates were washed five times with TBST between incubation steps. Finally, p-nitrophenyl phosphate (34045, Thermo Fisher Scientific) diluted in DE buffer (10.5 mM diethanolamine, 0.5 mM magnesium chloride; pH 9.8) was added for colour development, and absorbance was measured at 450 nm with a subtracted reference of 490 nm using a SpectraMax microplate reader (Molecular Devices).

### ELISPOT

MAIPN0B50 ELISPOT plates (Millipore) were coated with 2 µg ml^−1^ of anti-mouse IL5 or anti-mouse IFN-γ capture antibody and incubated overnight at 4 °C. On the next day, the plates were washed and blocked with 100 µl of 1% bovine serum albumin in PBS for 2 h at RT. Subsequently, 10^6^ mouse splenocytes were added to each well and stimulated with SARS-CoV-2 S1 (2 µg ml^−1^), SARS-CoV-2 N (2 µg ml^−1^, or AIM V media (negative control) overnight at 37 °C in 5% CO_2_. After 18 h of stimulation, the plates were washed and incubated with 2 µg ml^−1^ biotinylated anti-mouse IFNγ or IL5 diluted in 1% albumin/PBS for 1.5 h at RT. Next, 100 µl of alkaline phosphatase-conjugated streptavidin diluted 1:1000 in 1% albumin/PBS was added to each well, and the plates were incubated for 1.5 h at RT. Between each incubation step, plates were washed five times with PBST and two times with ddH_2_O. Finally, 100 µl BCIP/NBT substrate was added, and the plates were incubated at RT for spots to develop. The plates were then rinsed with ddH_2_O and dried at RT overnight. Spots were counted by an automated ELISPOT reader.

### Generation of pseudotyped SARS-CoV-2 and pseudovirus neutralization assay

Pseudotyped lentivirus with both luciferase and green fluorescent protein reporters was generated as described previously [14,15]. Specifically, HEK293T cells (in a 6-well plate, 60–70% confluent) were co-transfected with 0.34 µg of SARS-CoV-2-D614G-S-D19 WT [16] or Omicron S, 1 µg of lentiviral backbone-Luc2-ZsGreen [15] and 0.66 µg of packaging plasmid ps-PAX2 (12260, Addgene) plasmids using the JetPEI transfection reagent. The cell-culture medium was replaced at 16 hpt, and supernatants containing pseudotyped SARS-CoV-2 were collected at 48 and 72 hpt. The pseudotyped virus stocks were pooled, filtered through a 0.45-µm filter, aliquoted and stored at −80 °C.

Neutralization assay using pseudotyped SARS-CoV-2 was performed as described previously with minor modifications [15,17, 18]. Specifically, 96-well cell culture plates were coated with poly-l-lysine and seeded with ~5×10^4^ ACE2-expressing HEK293 cells per well. On the next day, serum samples from mice in the same vaccination groups were pooled and diluted 1:20 in D10 medium, incubated with the pseudotyped Wuhan or Omicron SARS-CoV-2 for 1 h at 37℃ and then utilized to infect the HEK293-ACE2 cells. Polybrene was added to a final concentration of 5 µg ml^−1^ to facilitate pseudotype virus infection by minimizing charge-repulsion between the virus and cells [19]. The infected cells were harvested at 48 h post-infection, and luciferase assay was performed using Luciferase™ Reporter Assay System (Promega) in a GloMax 20/20 Luminometer according to the manufacturer’s instructions.

### Viral neutralization assay

Vero76 cells were plated in 96-well flat-bottomed plates and maintained at 37 °C in a 5% CO_2_ incubator. On the next day, heat-inactivated mouse serum was serially diluted in 50 µl of DMEM and incubated for 1 h with 50 µl of DMEM containing 412 TCID_50_ of Wuhan SARS-CoV-2 or 822 TCID_50_ of Omicron SARS-CoV-2 infectious particles per well at 37 °C. The virus-serum mixture was then added to a monolayer of Vero76 cells and incubated for 1 h at 37 °C. Subsequently, 100 µl of DMEM supplemented with 2% FBS, 100 units penicillin and 100 µg streptomycin ml^−1^ was added to each well, and the samples were incubated for 2 days. All samples were run in duplicate, and the titre of neutralizing antibody (Nab) for each sample is determined as the reciprocal of the highest dilution with no cells showing cytopathic effects.

### Statistical analysis

Data analysis was performed using GraphPad Prism 9, and statistical differences were determined by non-parametric Mann–Whitney test. Data were expressed as mean±sem values, and statistical significance was demonstrated as follows: * if *P*<0.05 and ** if *P*<0.01.

## Results

### SARS-CoV-2 VLP generation and characterization

**Fig. 1.**
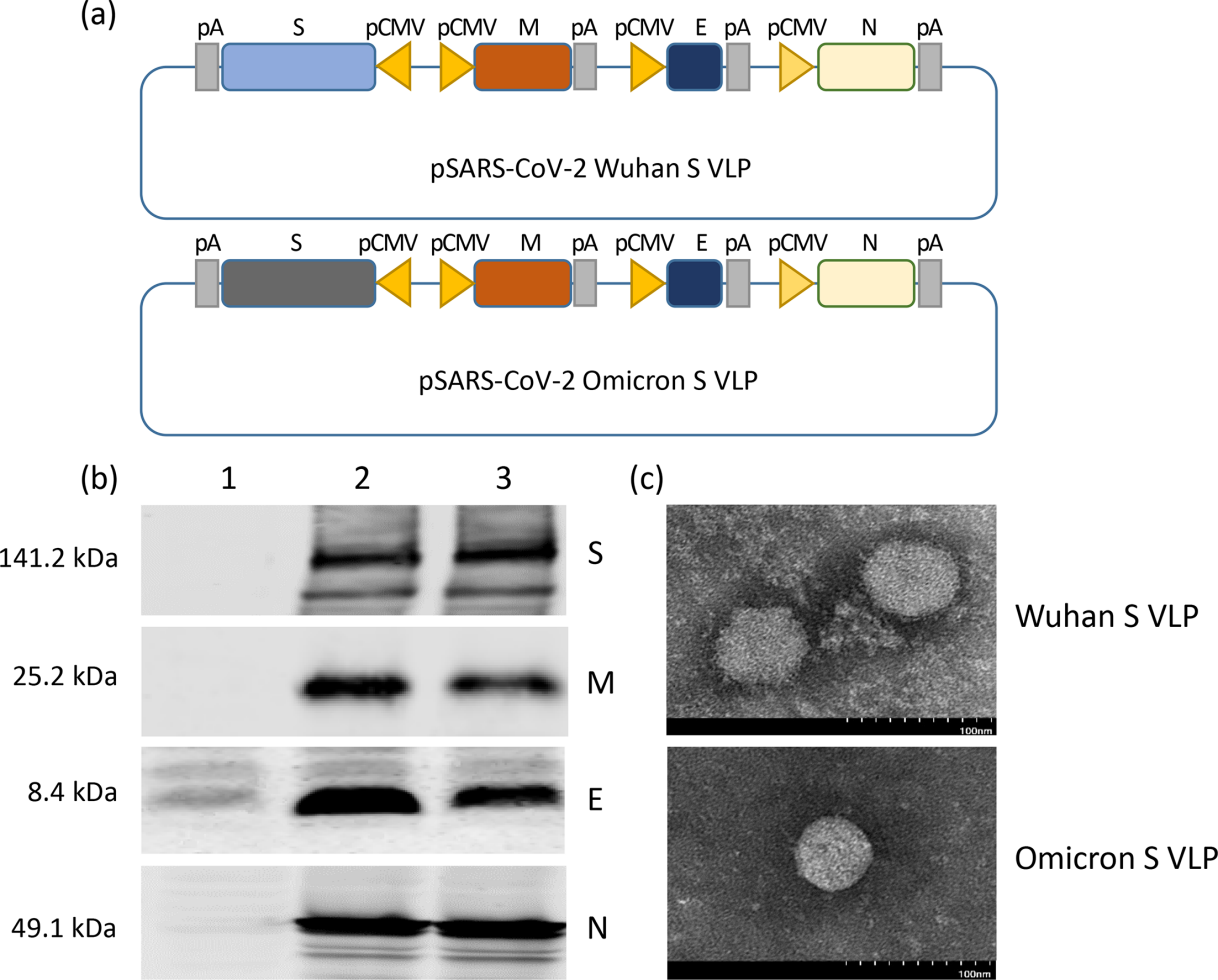
Construction and characterization of SARS-CoV-2 VLPs. (**a**) Schematic diagram of the SARS-CoV-2 VLP-expressing plasmids – pSARS-CoV-2-VLP1 and pSARS-CoV-2-VLP2 carrying spike gene of SARS-CoV-2 Wuhan and Omicron variant, respectively. S, M, E, and N, SARS-CoV-2 spike, membrane, envelope, and nucleocapsid genes, respectively; pA, poly A sequence. (**b**) Expression of SARS-CoV-2 spike, membrane, envelope, and nucleocapsid proteins in SARS-CoV-2-VLP-transduced HEK293 cells. (**c**) Transmission electron microscopic image of SARS-CoV-2 VLPs produced in HEK293 cells.

We cloned the genes encoding SARS-CoV-2 spike (S) of Wuhan or Omicron variant, membrane (M), envelope (E) and nucleocapsid (N) proteins of SARS-CoV-2 Wuhan strain into a baculovirus pBacMam vector as previously described (Fig. 1a) [20]. The expression of viral proteins after transduction with the recombinant BacMam was confirmed by protein-specific antibodies in Western blotting (Fig. 1b). The yields of purified SARS-CoV-2 VLP1 and VLP2 were 17.0 and 12.5 µg per 1×10^7^ transfected HEK293 cells, respectively. Transmission electron microscopic images in Fig. 1(c) showed that the morphology of our VLPs was very similar to that of authentic SARS-CoV-2 particles [21]. These results demonstrated the formation of SARS-CoV-2 VLP particles by the constructed BacMam. These VLPs were purified as described [20] and stored for less than 3 months before they were used in the immunization study.

### Immunogenicity of SAR-CoV-2 VLP antigens in mice

**Fig. 2.**
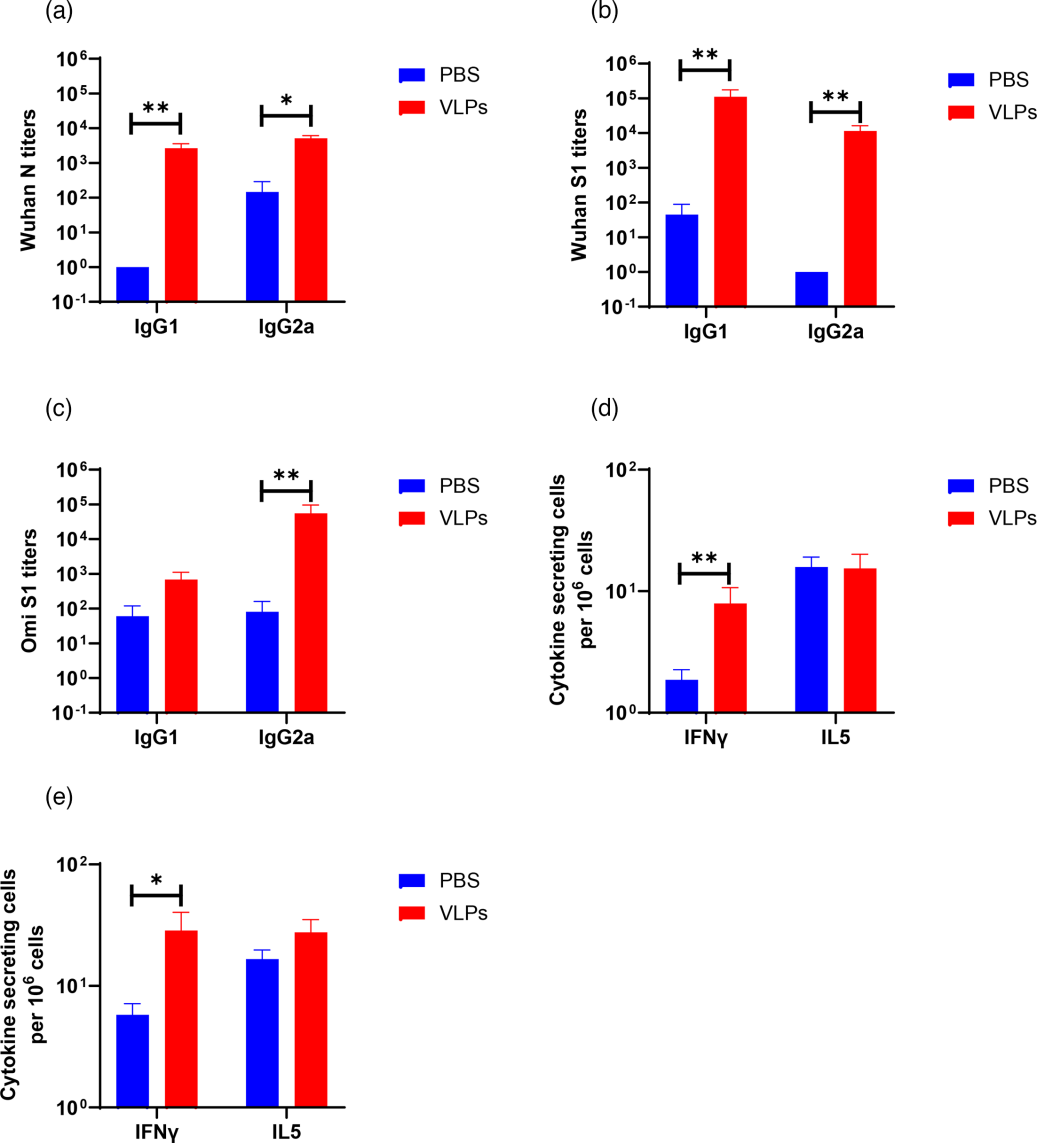
SARS-CoV-2-specific humoral and cellular immune responses in mice vaccinated with VLPs. Serum IgG1 and IgG2a litres against (**a**) Wuhan nucleocapsid, (**b**) Wuhan spike S1 and (**c**) Omicron spike S1 protein. Numbers of IFNγ and IL5 secreting splenocytes determined in response to *in vitro* restimulation with Wuhan (**d**) S1 protein and (**e**) N protein. Mice were immunized with VLPs containing Wuhan spike protein and boosted after 3 weeks with the same dose of VLPs containing the Omicron spike protein vaccine. Control groups were immunized with PBS. At 3 weeks after the second immunization, mice were euthanized to collect blood for ELISA and spleen for ELISPOT assays. ELISA litres are expressed as the reciprocal of the highest dilution, resulting in a value of two sd above the negative control serum. Cytokine-secreting cell numbers are expressed as the difference in the number of spots between protein-stimulated wells and medium-control wells. Statistical analysis was performed using the non-parametric Mann–Whitney test. Bars indicate the mean with sem. *P<0.05; **P<0.01.

To evaluate the immunogenicity of the SARS-CoV-2 VLPs in a prime-boost strategy, BALB/c mice were first vaccinated intramuscularly with 2.5 µg VLP1 and then boosted with 2.5 µg VLP2 3 weeks later. Animals were euthanized 3 weeks after the second vaccination, and their serum and spleen were collected for immunoassays. To evaluate the systemic immune responses, Wuhan N-, S1- and Omicron S1-specific IgG1 and IgG2a antibody levels were measured in the sera by ELISA (Fig. 2a, b and c). Significantly higher Wuhan N- and S1-specific IgG1 and IgG2a titres were observed in mice immunized with VLPs than in those immunized with PBS. In addition, mice immunized with VLPs produced significantly higher Omicron S1-specific IgG2a antibody titre than the PBS group. Although immunized mice generated elevated levels of Omicron S1-specific IgG1 antibody compared to the PBS group, the difference was not statistically significant. These results showed that VLPs promoted a balanced or Th1-type systemic humoral immune response.

To investigate the phenotype of the cellular immune response, Wuhan N- and S1-induced secretion of IFNγ and IL5 by splenocytes was measured by ELISPOT. Mice vaccinated with the VLPs induced significantly high IFNγ-secreting cells compared to PBS control, whereas IL5-secreting cells showed no significant difference (Fig. 2d, e), regardless of stimulation with Wuhan S1 or N protein. These results suggested that vaccination with VLPs elicited a Th1-biassed cell-mediated immune response.

### Neutralizing activities induced by the SARS-CoV-2 VLP antigens

**Fig. 3.**
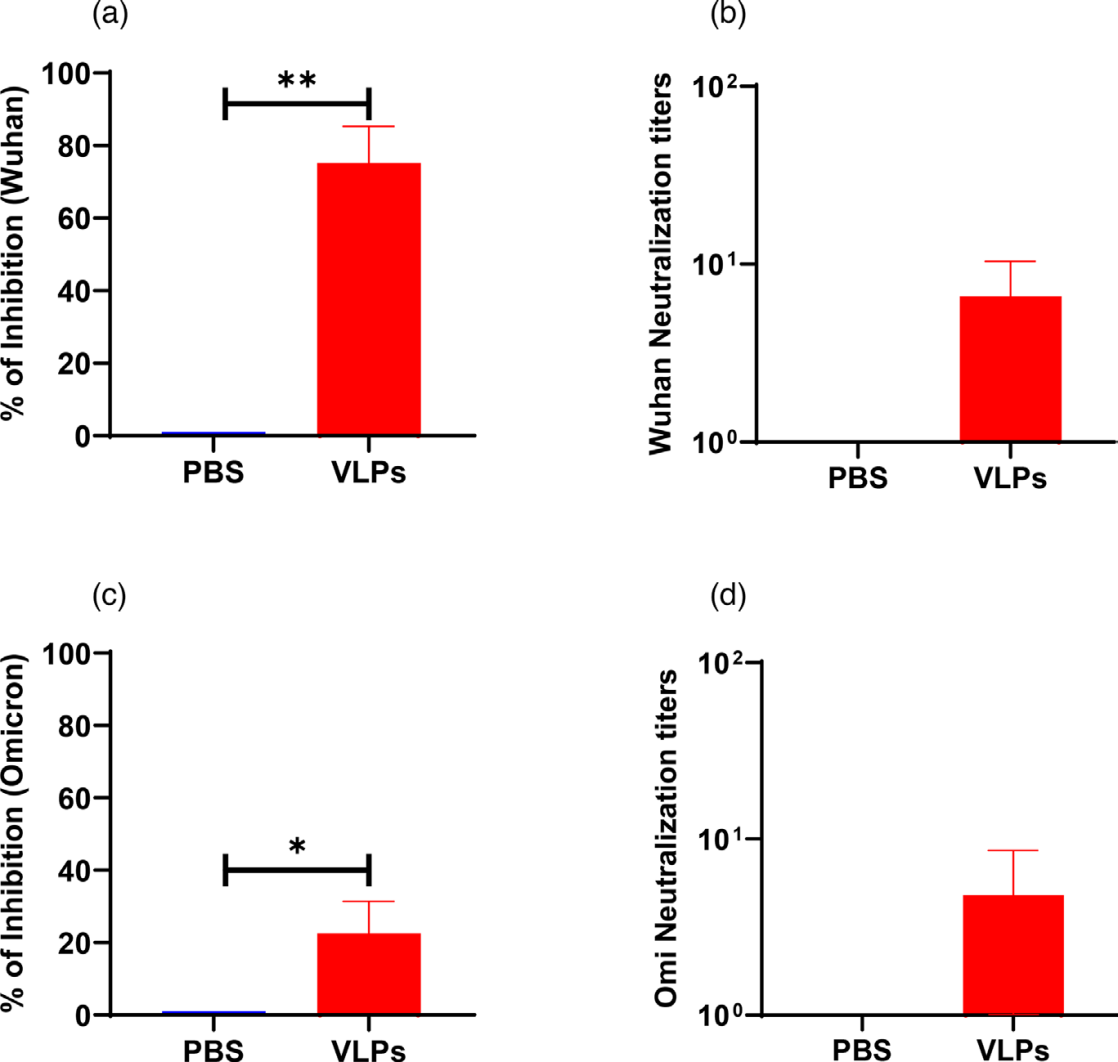
Serum neutralization activity of SARS-CoV-2 VLP-vaccinated mice. Relative luciferase units in cells infected with (**a**) Wuhan and (**c**) Omicron pseudovirus pre-treated with vaccinated mice sera. Pseudovirus was treated with sera from mice vaccinated with PBS, and VLPs, and then infected HEK293-ACE2 cells. The infected cells were harvested at 48 h post-transduction for determination of the neutralization activity by luciferase assay. Neutralization litres of vaccinated-mice sera against live (**b**) Wuhan and (**d**) Omicron variant. Mice were immunized as described in the legend for Fig. 2. Statistical analysis was performed using the non-parametric Mann–Whitney test. Bars indicate the mean with sem. *P<0.05; **P<0.01.

The biological function of the S1- and N-specific serum antibodies and virus-neutralizing antibody litres were determined by both pseudovirus and live virus-neutralization assays. When pseudotyped lentiviruses with the luciferase reporter were used in the neutralization assay, the VLP-vaccinated animals showed a significantly higher reduction of luciferase activities than the PBS group against both Wuhan and Omicron variants (Fig. 3a, c). Wuhan and Omicron-specific neutralization litres were also determined by viral neutralization assay using live viruses. Once again, mice immunized with VLPs developed higher neutralizing antibody litres than PBS-immunized animals against both the Wuhan and Omicron variants (Fig. 3b, d). No neutralizing antibodies were detected in the saline group. These results demonstrated that vaccination with VLPs produced Wuhan- and Omicron-specific neutralizing antibodies.

## Discussion

In a short amount of time, the highly transmissible SAR-CoV-2 virus caused the COVID-19 pandemic, which spread quickly over the world, infecting hundreds of millions of people and killing millions [1]. The global spread of this virus prompted an urgent demand for prevention efforts. Nonetheless, the SAR-CoV-2 virus continuously mutates, much like other RNA viruses, which causes new variants over time. As a result, significant effort has been made to develop new vaccines and improve the efficacy of existing vaccines. As a platform technology, recombinant baculoviruses are used to express either a single vaccine antigen or multiple antigens due to their superior safety profile, ability to induce balanced immune responses often without the need for adjuvants and scalability to manufacture production [22]. In this study, we used this technology to generate virus-like particles consisting of four structural proteins of SARS-CoV-2 and tested their immunogenicity.

Induction of a Th1 type or balanced immune response via vaccination is often necessary for vaccine protection against viral infection. Cell-mediated immunity plays an important role in antiviral processes [23]. Previously, it has been shown that the Th1 response plays a major role in influenza virus clearance and viral-specific IgG2a plays a critical role against influenza infection [24]. Another study showed that IgG2a antibody expression was associated with enhanced protection and viral clearance in animals vaccinated with influenza virus haemagglutinin-expressing viral replicon particles [25]. In our study, animals vaccinated with VLPs induced high IgG2a litres. Recent research suggests that neutralizing antibodies may act as a correlate of vaccine protection against SARS-CoV-2 in people. Mice vaccinated with VLPs in our study demonstrated viral neutralizing activity.

Overall, our findings demonstrated that VLP produced strong humoral and cellular immune responses, indicating that it could protect against SARS-CoV-2 infection and serve as a potential vaccine candidate. The VLP approach may be used and refined in the future to create multivalent vaccines that will protect against future infectious pathogens.

## Abbreviations

CMV: cytomegalovirus
COVID-19: coronavirus disease 2019
DMEM: Dulbecco’s Modified Eagle Medium
hpt: hours post-transfection
RT: room temperature
SARS-CoV-2: severe acute respiratory syndrome coronavirus 2
TBST: tris-buffered saline with 0.1% tween 20
TSE: Tris/Saline/EDTA
VLPs: virus-like particles
